# Comparison between the Traditional Landmark Method and the Real-Time Ultrasound Technique to Determine the Correct Depth of Insertion of Central Venous Catheter in Right Jugular Vein in Pediatric Neurosurgical Patients: A Randomized Controlled Trial

**DOI:** 10.7759/cureus.117929

**Published:** 2026-10-14

**Authors:** Shagun Paliwal, Brijesh P Singh, Ravi Prakash, Premraj Singh, Vinita Singh

**Affiliations:** 1 Anaesthesiology, King George's Medical University, Lucknow, IND; 2 Anaesthesiology and Critical Care, King George's Medical University, Lucknow, IND

**Keywords:** central venous catheterization, insertion depth, internal jugular vein, landmark technique, paediatric neurosurgery, ultrasound guidance

## Abstract

Background: Central venous catheterization (CVC) is essential in paediatric neurosurgical patients for monitoring and therapeutic purposes. However, determining the optimal catheter insertion depth remains a challenge due to anatomical variability and a lack of standardized guidelines. This study aimed to compare the effectiveness of a height-based ultrasound (USG)-guided technique versus the traditional anatomical landmark approach for right internal jugular vein (IJV) catheterization in children.

Methods: In a prospective randomized controlled trial conducted at a tertiary care centre in North India, 126 children aged 4-12 years undergoing elective neurosurgery were randomized into two groups: Group A (landmark technique) and Group B (USG-guided, height-based formula). Outcomes assessed included insertion depth accuracy, number of attempts, insertion time, and complications.

Results: The USG group demonstrated significantly greater first-attempt success (85.7% vs. 66.7%; *p* = 0.037) and more accurate catheter placement (variation from carina: 2.65 ± 2.60 mm vs. 3.93 ± 3.78 mm; *p* = 0.028). Mean insertion depth was longer in the USG group (8.82 ± 1.86 cm vs. 8.17 ± 1.51 cm; *p* = 0.036), and insertion time was also higher (19.98 ± 3.89 min vs. 16.06 ± 3.98 min; *p* < 0.001). Although not statistically significant, complication rates were lower in the USG group.

Conclusion: Height-based USG guidance offers a more accurate and effective method for paediatric IJV CVC placement, improving first-attempt success and reducing malposition without increasing complications. Despite a modest increase in procedural time, USG should be considered the preferred technique in this population.

## Introduction

Central venous catheterization (CVC) is a very common practice among paediatric patients undergoing neurosurgical procedures, as it enables the monitoring of central venous pressure, fluid balance, administration of large volumes of fluids, blood products, and drugs, obtaining blood for laboratory investigations, and keeping track of hemodynamic status [1-3]. Though securing central venous access is one of the fundamental interventions in paediatric anaesthesia, getting it done properly in small children is challenging due to anatomical factors such as thin and delicate veins and excess subcutaneous fat, which makes visualization and palpation of veins difficult [4].

Traditionally, the anatomical landmark approach is used for internal jugular vein (IJV) cannulation, but it is a highly skill-based procedure. The emergence and increased accessibility of ultrasonography (USG), which has demonstrated better outcomes in terms of success rate, time efficiency, and safety, has made this technique popular for CVC cannulation [5-8]. However, CVC cannulation carries well-known risks of life-threatening mechanical complications like cardiac perforation, tamponade, and cardiac arrhythmia. Positioning of the catheter tip near the superior vena cava and right atrium junction decreases the incidence of these complications [9-11]. The carina is a common radiographic target level for the CVP tip position [12].

For paediatric patients, accurate guidelines are not available for optimal insertion depth of CVC. There have been several studies regarding optimal depth of central venous cannulation. Common methods used to decide a proper insertion depth of CVC include the Peres formula, the Andropoulos formula, right atrial ECG, and radiographic guidance on transesophageal echo (TOE). The puncture site at the lower part of the neck may cause catastrophic deep insertion, whereas the higher approach for CVC cannulation may produce a shallow insertion. The CVC tip should not be in or allowed to migrate into the heart to prevent disastrous complications that carry a high risk of mortality. Estimating the depth of central venous catheter insertion based solely on patient characteristics, such as age, may be insufficient because anatomical variations in the neck can influence the venous puncture site and the required insertion depth [13-19]. In general, we have a traditional landmark approach that uses prominent landmarks like the right clavicular notch and second intercostal space. Several studies have found that a height-based formula overcomes variation in anatomical structure and identifies optimal depth [20-27].

Hence, the present study was planned to determine whether a USG-guided height-based formula could help to determine the optimal depth of insertion, reduce cannulation time, reduce complication rate, and reduce the number of attempts of CVC cannulation as compared to the landmark technique among pediatric neurosurgical patients at a tertiary care hospital.

## Materials and methods

This prospective, single-blind (outcome assessor blinded to group allocation), randomized controlled trial was conducted in the Department of Anaesthesiology at King George’s Medical University, Lucknow, Uttar Pradesh, India, over a 15-month period from January 2023 to March 2024. The study was approved by the Institutional Ethics Committee of King George's Medical University (approval number: IEC/2358/Ethics/2023) and prospectively registered with the Clinical Trial Registry of India (registration number: CTRI/2023/09/057810). Prior to participation, written and informed consent was secured from the parents or legally authorized representatives of all enrolled children.

Study population

Sample Size

The required sample size was determined using data from earlier published literature, assuming a moderate effect size of 0.5 for differences in optimal CVC tip depth between the two techniques [18]. Applying the formula $N = \frac{2 \times (Z_{\alpha} + Z_{\beta})^2}{ES^2}$, with Zα = 1.96 for a 95% confidence level, Zβ = 0.842 corresponding to 80% statistical power, and an effect size (ES) of 0.5, a total of 126 patients were calculated to be necessary, with equal allocation to each study arm.

Eligibility Criteria

Children aged between four and 12 years, of either sex, American Society of Anesthesiologists (ASA) physical status I-III, and scheduled for elective craniotomy under general anesthesia were included. Patients were excluded if they had a history of neck trauma or surgical scarring, cardiac rhythm abnormalities, bleeding disorders, congenital malformations, or if consent was declined.

The CONSORT (Consolidated Standards of Reporting Trials) flow diagram is shown in Figure 1.

**Figure 1:**
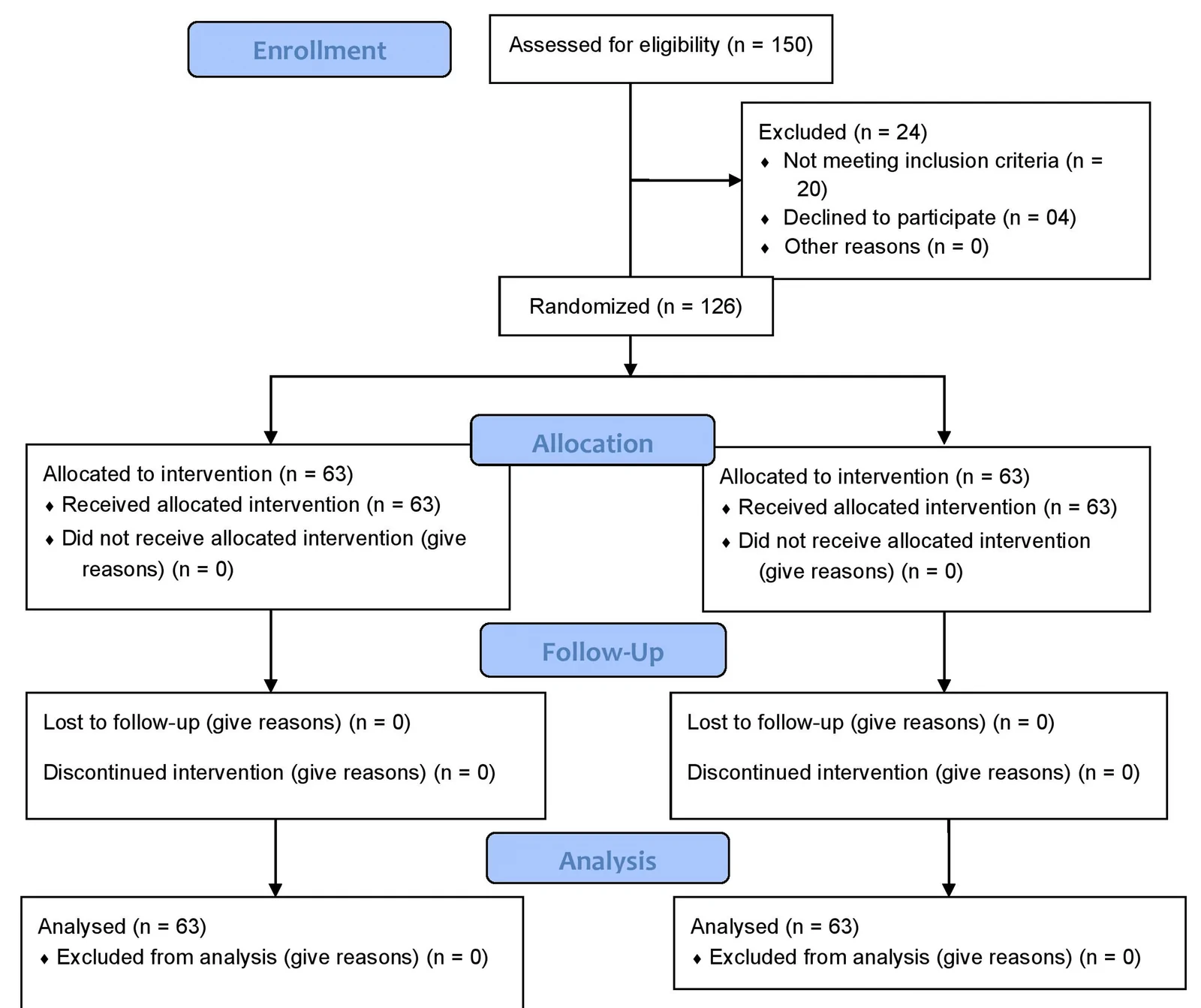
CONSORT flow diagram CONSORT: Consolidated Standards of Reporting Trials

Randomization and procedure

Participants were randomly assigned to one of two groups using a computer-generated randomization sequence. In Group A, the depth of CVC (Certofix® Trio, triple-lumen, 4-6 Fr, 8-15 cm; B. Braun Melsungen SE, Melsungen, Germany) insertion was estimated using an anatomical landmark-based approach, calculated as the sum of the distance from the venous entry site to the right clavicular notch and the distance from the clavicular notch to the carina (second intercostal space) [28]. In Group B, catheter depth was calculated using a height-derived formula under real-time USG guidance, expressed as depth (cm) = 0.062 × height (cm) + 2.2438 [29]. Figure 2 shows the clinical illustration of the two methods.

**Figure 2:**
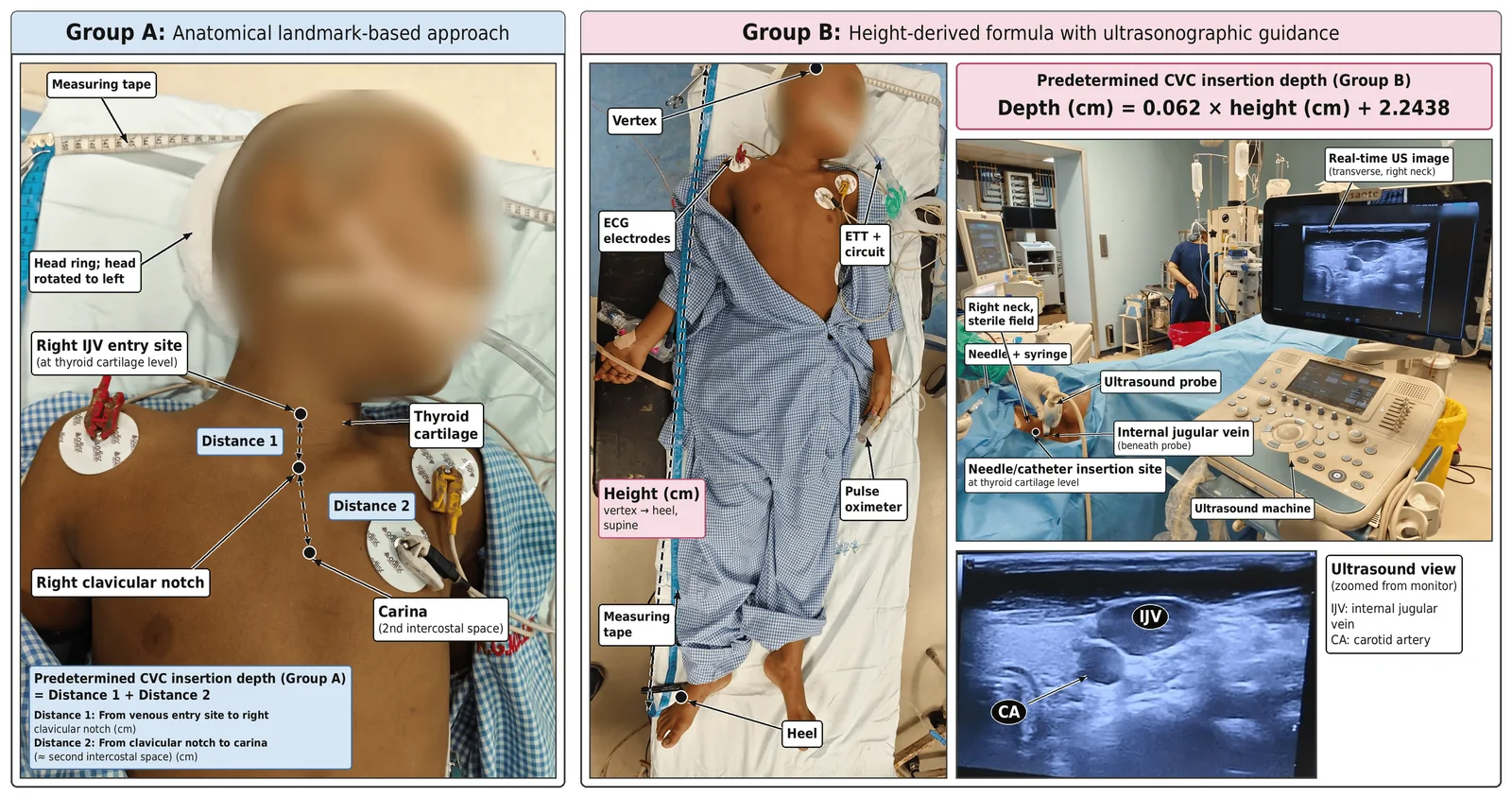
Clinical illustration of the two methods for predetermining right internal jugular vein CVC insertion depth CVC: central venous catheterization; ETT: endotracheal tube; US: ultrasound

Standard monitoring such as ECG, non-invasive blood pressure (NIBP), peripheral oxygen saturation (SpO2), end-tidal carbon dioxide (EtCO2), and temperature monitoring was done in all patients. Intravenous access was secured, and anesthesia was induced and maintained according to a standardized institutional protocol.

For central venous access, patients were placed in a 10-15° Trendelenburg position with shoulder elevation achieved using a towel roll. Following strict aseptic preparation, the right IJV was cannulated at the level of the thyroid cartilage. The predetermined insertion depth, based on group allocation, was followed, and the final catheter tip location was confirmed intraoperatively using C-arm fluoroscopy. A catheter tip position lying within 1 cm proximal or distal to the carina was defined as optimal placement. Positions exceeding this range were categorized as either under-inserted or over-inserted and labeled suboptimal. In such cases, catheter repositioning was performed before commencement of surgery.

All procedures were carried out by a single experienced anesthesiologist to eliminate operator variability. Cannulation time was defined as the interval between skin puncture and successful aspiration of venous blood after guidewire removal. Up to three attempts were permitted, beyond which the procedure was considered unsuccessful.

The primary endpoint of the study was the accuracy of CVC insertion depth. Secondary endpoints included the number of cannulation attempts, occurrence of arrhythmias, and procedure-related complications.

Data analysis

Data analysis was performed using IBM SPSS Statistics for Windows, version 25.0 (IBM Corp., Armonk, New York, United States). Continuous variables were recorded as mean ± standard deviation (SD) and analyzed by independent samples t-test. Categorical data were compared using the Chi-square test or Fisher’s exact test, as appropriate. Statistical significance was defined as a p-value <0.05.

## Results

A total of 126 patients were enrolled in the study and equally allocated to two groups (n=63 each). The depth of insertion of CVC in the right IJV was determined in Group A using the anatomical landmark method, and in Group B using a height-based formula under real-time USG guidance. Baseline demographic and clinical characteristics were comparable between the two groups (Table 1). CVC cannulation attempts, insertion depth and time, mean insertion depth, mean insertion time, and complications are listed in Table 2.

**Table 1:** Baseline demographics and clinical characteristics

Characteristics	Group A (n=63)	Group B (n=63)	Test	P value	
Age (years), mean±SD	7.10±2.53	7.21±2.71	Student-t test	t=0.221	p=0.826
Height (cm), mean±SD	106.8±22.98	107.05±29.92	Student-t test	t=0.034	p=0.965
Weight (kg), mean±SD	34.43±10.27	34.96±10.91	Student-t test	t=1.345	p=0.181
Gender, n (%)	-	-	Chi-sq. test	χ2=0.034	p=0.854
Male	39 (61.9%)	40 (63.5%)	-	-	-
Female	24 (38.1%)	23 (36.5%)	-	-	-

**Table 2:** Intergroup comparison of CVC procedural outcomes CVC: central venous catheterization

S. No.	-	Group A (n=63)	Group B (n=63)	Test	P value	
Cannulation Attempts				Chi-sq. test	χ2=8.470	p=0.037*
1.	One	42 (66.7%)	54 (85.7%)		-	-
2.	Two	14 (22.2%)	8 (12.7%)	-	-	-
3.	Three	5 (7.9%)	0 (0%)	-	-	-
4.	Failure	2 (3.2%)	1 (1.6%)	-	-	-
Time Duration		16.11±4.03	19.98±3.89	Student-t test	t=5.418	p<0.001*
Insertion Accuracy				Chi-sq. test	χ2=10.214	p=0.006*
1.	Under-insertion	7 (11.1%)	0 (0%)	-	-	-
2.	Over-insertion	0 (0%)	3 (4.7%)	-	-	-
3.	Optimum insertion	54 (85.7%)	59 (93.6%)	-	-	-
Depth		8.17±1.51	8.82±1.86	Student-t test	t=2.126	p=0.036*
Complications				Chi-sq. test	c^2^=2.125	p=0.547
Intra-operative						
1.	Hematoma	6 (9.52%)	4 (6.35%)	-	-	
2.	Arrhythmias	2 (3.17%)	2 (3.17%)	-	-	
3.	Arterial Puncture	3 (4.76%)	0 (0%)	-	-	
Post-operative						
1.	Pneumothorax	2 (3.17%)	1 (1.59%)	-	-	

## Discussion

CVC is essential in paediatric neurosurgical anaesthesia for monitoring and therapeutic purposes. Securing vascular access in children often poses technical challenges due to small-calibre veins, delicate vessel walls, and increased subcutaneous fat, which collectively impair vein palpation and visualisation [4]. CVC carries a risk of serious mechanical complications, including cardiac perforation, tamponade, and arrhythmias, often due to malpositioning of the catheter tip [9-11]. In paediatric patients, the absence of standardised guidelines for CVC insertion depth poses challenges, as traditional formulas may not account for anatomical variability. Height-based and USG-guided approaches have emerged as more accurate alternatives, improving placement accuracy and reducing complications [13,14,18,19,21,22,26,27]. In our study, we compared the height-based USG-guided depth of insertion of CVC with the conventional anatomical landmark technique in paediatric neurosurgical patients. 

Both groups in our study demonstrated low and comparable rates of intraoperative and postoperative complications, including haematoma, arterial puncture, arrhythmia, and pneumothorax. These findings are consistent with those of Yang et al. [6] and Dolu et al. [8]. They also reported low complication rates without significant differences between techniques. Some studies suggest a lower incidence of complications, particularly arterial puncture, with USG guidance [21].

Limitations of the study included a relatively small sample size and a specific age group of participants. Future research should focus on larger, randomized controlled trials encompassing all paediatric age groups. Comparative evaluation of USG-guided CVC insertion using both anatomical landmarks and height-based formula-derived depth estimations may offer more robust insights into optimal catheter placement.

## Conclusions

In this study of paediatric patients undergoing neurosurgical procedures, the height-based USG guidance technique for CVC insertion demonstrated superior accuracy in achieving optimal insertion depth and higher first-attempt success rates than the anatomical landmark technique. Although the height-based USG guidance technique required longer insertion time, it resulted in fewer attempts and comparable complication rates. Notably, the anatomical landmark technique was associated with a higher risk of under-insertion, whereas the height-based USG guidance technique showed a tendency for slight over-insertion. Larger randomized controlled trials are warranted to further validate these results. 
